# Composition of Primary and Secondary Metabolite Compounds in Seeds and Pods of Asparagus Bean (*Vigna unguiculata* (L.) Walp.) from China

**DOI:** 10.3390/molecules25173778

**Published:** 2020-08-19

**Authors:** Irina Perchuk, Tatyana Shelenga, Maria Gurkina, Elena Miroshnichenko, Marina Burlyaeva

**Affiliations:** 1 N.I. Vavilov All-Russian Institute of Plant Genetic Resources 42,44, B. Morskaya Street, 190000 Saint-Petersburg, Russia; t.shelenga@vir.nw.ru (T.S.); m.burlyaeva@vir.nw.ru (M.B.); 2 Astrakhan Experiment Breeding Station, Branch of N.I. Vavilov All-Russian Institute of Plant Genetic Resources, village Yaksatovo, 416162 Astrakhan Region, Russia; m.gurkina-08@mail.ru (M.G.); aos_vir@mail.ru (E.M.)

**Keywords:** asparagus bean, *Vigna unguiculata*, old Chinese landraces, metabolomic profile, seed, pod

## Abstract

Asparagus bean immature pods and seeds are popular as food products for healthy and functional nutrition. Gas chromatography with mass spectrometry was used to compare metabolomic profiles of seeds and pods yielded by old Chinese landraces and the modern cultivars ‘Yunanskaya’ and ‘Sibirskiy razmer’. About 120 compounds were identified. The content of a majority among groups of compounds was higher in pods than in seeds. The amount of free amino acids in pods was 47 times higher, polyols and phytosterols 5 times higher, phenolics 4 times higher, and organic acids and saponins 3 times higher than in seeds. Differences were found in the relative content of compounds. Among phenolic compounds, the dominant one for seeds was protocatechuic acid, and for pods 4-hydroxycinnamic acid. Only polyols were identified in seeds, but pods additionally contained ethanolamine, phytol, and phytosphingosine. The ratio for nonsaturated/saturated fatty acids was 2.2 in seeds and 1.4 in pods. Seeds contained more stigmasterol, and pods more β-sitosterol. Aglycones of saponins were identified: cycloartenol in seeds, α- and β-amyrins in pods. Oligosaccharides dominated in both seeds and pods. Landraces manifested higher protein content in pods, while modern cultivars had pods with higher contents of organic acids, polyols, monosaccharides, and fatty acids. The results obtained confirm the high nutritional value of asparagus bean seeds and pods, and the prospects of their use in various diets.

## 1. Introduction

The asparagus bean, or yardlong bean (*Vigna unguiculata* (L.) Walp., culivar group *sesquipedalis*), is a valuable vegetable cowpea crop cultivated since long ago in the Southeast Asia. Its center of origin is China [1]. In Asian countries, it is numbered among the 10 staple vegetable crops. In Russia, in the southern areas of Primorsky Territory adjacent to China and Korea, the history of vegetable cowpea cultivation by the local population has lasted for more than a century. In the regions where asparagus bean has been traditionally cultivated, it is grown for the sake of young leaves, juicy immature pods, and dry seeds, and used for food like green common beans. Presently, asparagus bean is widespread across many counties in Europe, Africa, and America [2,3]. The crop owes its popularity over the world to its immature pods, distinguished for their excellent taste and technological qualities, reaching, in some cultivars, 1 m in length.

Seeds of *V. unguiculata* are characterized by their high content of proteins, carbohydrates, trace elements and macrominerals, low lipid content, and presence of bioactive compounds. Sufficiently extensive information has been accumulated in past years about antioxidant, anticancer, antibacterial, and other activities of peptides, phenolic compounds, phytosterols, and other substances contained in the seeds of this species [4,5,6]. Because of such composition, the crop has become widely known as a source of healthy dietary and functional nutrition [7,8,9,10,11].

Despite the fact that pods, seeds, and young leaves of *V. unguiculata* are used for food, only seed qualities are analyzed in a majority of scientific publications. The data describing the nutritional value of other plant parts are rather limited. Development of improved vegetable cultivars of asparagus bean require as much comprehensive information as possible about the biochemical composition of immature pods in various genotypes of the crop.

VIR preserves in its collections old local varieties of *V. unguiculata* from China, collected in the early 20th century and notable for their utmost genetic diversity. Landraces serve as a rich reserve of useful traits, often lost in modern commercial cultivars. Comparative analysis of the biochemical compositions in seeds and pods of asparagus bean landraces and modern cultivars would make it possible to identify the range of variation for nutrients, including bioactive ones, and discover the effect of the breeding process on these indicators. We have failed to find publications dedicated to specialized research into the biochemical composition of immature pods and seeds of asparagus bean.

Along with the classical techniques of biochemical analysis, the modern methods of chromatography combined with mass spectrometry (LC-MS and GC-MS) are effective in obtaining the most comprehensive information on metabolites of potential nutritional sources [12,13].

In view of the above-mentioned, the aim of this work was to make a comparative analysis of the total protein content and GC-MS metabolomic profiles in mature seeds and immature pods of Chinese landraces and modern Russian cultivars (developed on the basis of genotypes from China) of asparagus bean from the VIR collection.

## 2. Results and Discussion

**Figure 1:**
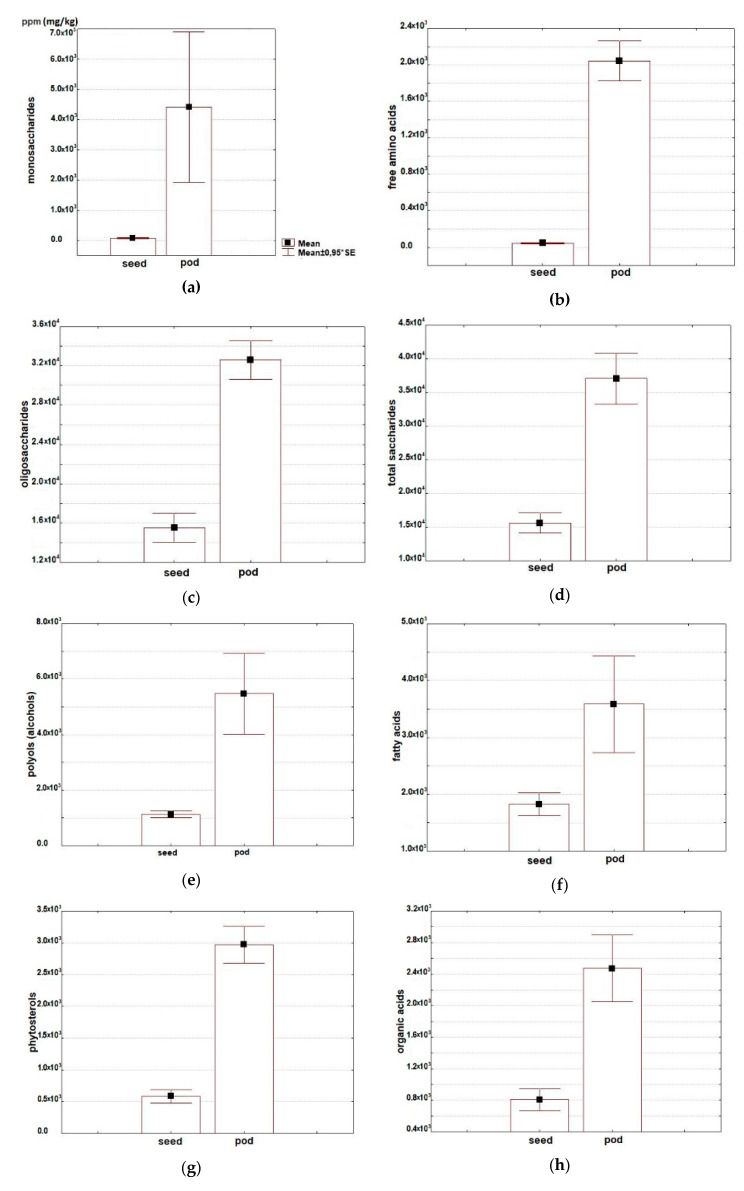

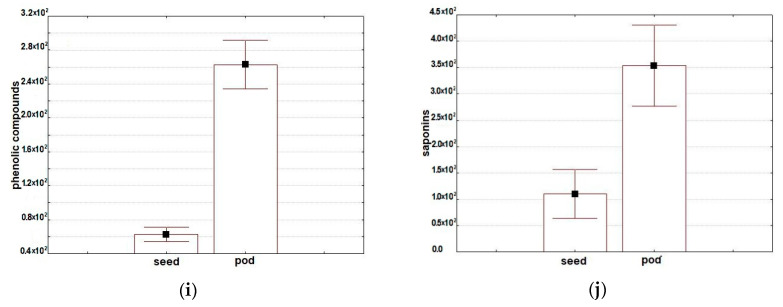
Average content of (**a**) monosaccharides, (**b**) free amino acids, (**c**) oligosaccharides, (**d**) total saccharides, (**e**) alcohols, (**f**) free fatty acids, (**g**) phytosterols, (**h**) organic acids, (**i**) phenolic compounds, and (**j**) saponins in seeds and pods of *Vigna unguiculata*, (ppm/dry matter).

The research has shown a considerable variability in the major groups of primary and secondary metabolite compounds in asparagus bean seeds and pods (Table 1; Figure 1).

### 2.1. Total Protein Content

Total protein content in seeds of the studied accessions varied from 26.8% to 27.5%, in pods from 29.0% to 33.5% (Table 1). According to the published data, asparagus bean is characterized by significant genetic diversity of protein parameters in seeds and pods. Protein concentrations in seeds vary from 15.1% to 38.5%, in pods from 17.7% to 23.1% [14,15,16]. The measured protein content in seeds complied with the results obtained by other researchers, but in pods it was higher in our experiment, which may be explained either by reproducing accessions under different climate conditions or by different levels of ripeness in the pods collected for testing. Most of the studies exploring protein content in asparagus bean are dealing with seeds, while there is practically no data concerning pods. The main protein fractions of asparagus bean seeds are globulins (up to 70% of the total content), albumins, and glutelins, with prolamins present on a small scale [15,16,17,18]. Such composition of the total protein is favorable for its good assimilation; cowpeas are an alternative source of plant protein for those allergic to soybean protein. The whole grain of cowpeas and protein isolates extracted from it reduce the cholesterol level in human and animal blood [19]. Such an effect is associated with β-vignin of the 7S globulin family present in seeds [17]. The hypocholesterolemic effect may be induced by a decrease of cholesterol absorption by the intestines and an increase of its excretion from the organism as well as the influence of some cowpea peptides on the expression of genes regulating lipid metabolism [5,19]. Legume bioactive peptides, 2–20 amino-acid residues in length, attract more and more attention [9]. When the processes undergoing in the gastrointestinal tract of mammals are simulated, cowpea seed protein extracts exposed to enzymatic hydrolysis demonstrate antioxidant, anticancer, and antidiabetic activities [4,5,20,21]. With extension of the seed germination time, protein extracts from germinating cowpea seeds have been observed to demonstrate increased activity. It is probably also associated with the appearance (generation) of bioactive peptides as a result of storage protein degradation under the impact of endopeptidase activity [22]. There are also published data on the antibacterial activity of one more fraction in cowpea seeds—11S globulins [23].

### 2.2. Analysis of Metabolomic Profiles in Seeds and Pods

The analysis of metabolomic profiles in seeds and pods resulted in identifying, in total, around 120 compounds representing major plant metabolite groups (Tables S1 and S2).

#### 2.2.1. Free Amino Acids

**Figure 2:**
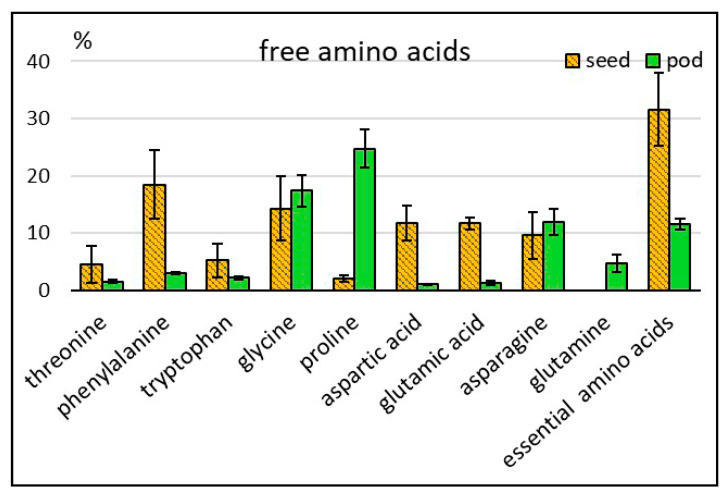
Relative content of some amino acids in asparagus bean seeds and pods (% of the total amount of this group of compounds).

Twenty-five free amino acids were identified in asparagus bean seeds and pods, including eight essential and six non-proteinogenic ones. The total amino acid content in pods (20433 ppm) considerably exceeded this indicator in seeds (433) (Table 1; Figure 1b). Low content of free amino acids in the flour produced from asparagus bean seeds was also observed by other researchers [16]. In this experiment, pods and seeds manifested differences in the qualitative composition as well: the set of amino acids in pods was more diverse. In relative units, however, the total percentage of essential amino acids in seeds was higher than in pods: 31.6 and 11.6% of their total content, respectively (Figure 2). Seeds differed from pods by a higher percentage of such essential amino acids as phenylalanine (18.5% and 3.0%, respectively), tryptophan (5.2% and 2.1%) and threonine (4.6% and 1.5%). High phenylalanine percentage in seeds was reported by other sources as well [17,18]. Phenylalanine plays an exclusive role in plant metabolism, serving as a nexus between primary and secondary metabolisms—the metabolism of aromatic amino acids and that of phenolics. Unlike seeds, asparagus bean pods contained methionine and arginine. Similarly to all other legumes, methionine in asparagus bean seeds is one of the limiting amino acids in protein. To increase the nutritional value, asparagus bean grain is used to produce protein isolates, wherein the content of methionine and arginine may be multiplied and reach the level of lysine content or even exceed it [8,24]. The most pronounced differences were observed in the total percentage of aspartic and glutamic acids, serving as the amino group donors: 23.4% in seeds, and 2.2% in pods. As for pods, the total percentage of asparagine and glutamine—one of the transport forms of nitrogen in plants—was 16.6%, while in seeds only asparagine was present (9.6%). A high percentage of proline (24.8%) was found in pods, compared with a much lower level in seeds (2.1%). Proline plays an important role in the regulation of osmotic processes at the cell level in plants. Asparagus bean pods were distinguished for the presence of ornithine, which participates, along with proline and arginine, in the plant’s response to environmental stressors [25]. Non-proteinogenic amino acids were represented by β-alanine, gamma -aminobutyric acid (GABA), hydroxyproline, norleucine, pipecolic acid, and oxoproline. All of them, with the exception of oxoproline, a structural element of cell membranes, were identified only in pods. Non-proteinogenic acids perform various functions in plants, including protection against environmental stressors [26,27]. They also play an important role in the metabolism of mammals. Pipecolic acid participates in the lysine metabolism, while β-alanine is a part of pantothenic acid which is incorporated into coenzyme A, one of the crucial metabolic compounds. Gamma-aminobutyric acid is a neurotransmitter in the human central nervous system.

#### 2.2.2. Saccharides

**Figure 3:**
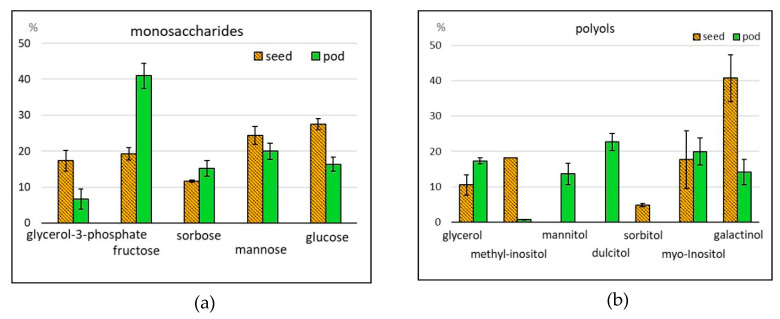
Relative contents of some (**a**) monosaccharides and (**b**) polyols in asparagus bean seeds and pods (% of the total amount of compounds in the given group).

High carbohydrate content is characteristic of cowpeas: up to 68% in seeds and pod valves, and up to 82% in young pods [19,28,29]. In this research, 15 compounds were identified, representing mono- and oligosaccharides and their derivatives. The total content of saccharides was 15,596 and 37,072 ppm in asparagus bean seeds and pods, respectively (Table 1; Figure 1a,c,d). With this, the percentage of oligosaccharides was 99.5% in seeds and 89.9% in pods. Pods contained more sucrose than seeds (31,519 versus 13,509 ppm), while seeds contained more raffinose (2013 versus 1083 ppm). The differences in monosaccharide content were more expressed. Respective levels for seeds and pods were 74 and 4409 ppm (Table 1, Tables S1 and S2). Monosaccharides were represented by hexoses (fructose, glucose, sorbose, galactose, mannose, rhamnose, and altrose), pentose (ribose) and triose (glycerol 3-phosphate). The greatest differences were recorded for the content of glycerol 3-phosphate, whose percentage in the total monosaccharides was 17.3% and 6.7% for seeds and pods, respectively, and fructose, 19.2% and 41.0% of the total saccharides content, respectively (Figure 3a). Glucosamine, methylglucoside, and methylgalactoside were absent in seed, but they were identified in pods. One of the constraints in legume produce consumption is flatulence, presumably associated with the presence of α-oligogalactosides—raffinose, stachyose, and verbascose [6,8,9]. These oligosaccharides, non-splitting in the small intestines, are regarded as antinutrients. At the same time, they are a substrate for the microbiota in the large intestines, i.e., prebiotics. In the process of seed germination or culinary processing, the percentage of raffinose and stachyose can become 80% lower; with this, the content of monosaccharides easily digestible by humans will increase [30,31].

#### 2.2.3. Alcohols

The total alcohols content in the studied accessions was 1127 ppm in seeds and 5678 in pods (Table 1; Figure 1e). Seeds contained only sugar alcohols and their derivatives; as for pods, in addition to the abovementioned compounds, there were amino alcohols: ethanolamine (65 ppm), phytosphingosine (37) and phytol (104) (Table S2). A considerable share in seeds (87.3%) and pods (72.4%) belonged to hexatomic sugar alcohols. In plants, this group of compounds may act as a respiratory substrate or storage compounds or participate in the protection against various stresses, etc. [32]. In this study, pods demonstrated the presence of dulcitol and mattinol, while seeds contained sorbitol. Seeds were observed to have high percentages of galactinol (40.8%) and the derivatives of inositol—myo-inositol, myo-inositol 2-phosphate, and methyl-inositol (41.7%) (Figure 3b). In pods, these indicators were 14.2% and 21.0%, respectively. Galactinol and myo-inositol in plants serve as intermediates for synthesis of the abovementioned α-oligo-galactosides [33]. Myo-inositol plays an important role in human metabolism, participating as a secondary messenger in regulating receptor functions in the cardiovascular, immunity and central nervous systems, sugar and lipid metabolism, etc. [34]. Phytic acid, a phosphorylated derivative of myo-inositol, and its salts perform an important function in plant metabolism. Dietitians, however, regard this acid as an antinutrient, because of its negative effect on the assimilation of proteins and minerals. The content of phytic acid in asparagus bean is significantly decreased by seed germination or culinary processing [35,36]. The attitude towards phytic acid as an antinutrient is not so unequivocal: recently, many publications have demonstrated its anticancer, antioxidant, antidiabetic activities, and other ‘useful properties’ [37]. It should be mentioned that phytic acid was not found in the asparagus bean accessions analyzed in the framework of this study.

#### 2.2.4. Free Fatty Acids

**Figure 4:**
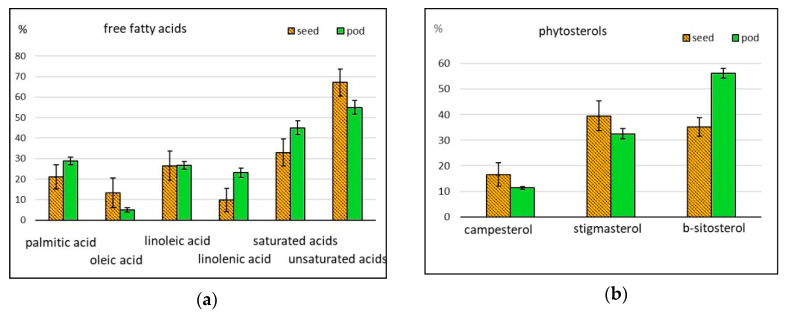
Relative content of some (**a**) free fatty acids and (**b**) phytosterols in asparagus bean seeds and pods (% of the total amount of compounds in the given group).

Cowpeas attract attention of dietitians because of their low fat (lipid or oil) content, ranging from 1.5% to 4.8%, and component composition [8,19]. The oil content in asparagus bean accessions grown in Russia was 1.6% in seeds, and 2.8% in pods [15]. We identified nine free fatty acids (FA) and their derivatives, their aggregate content in seeds and pods being 1827 and 3588 ppm, respectively (Table 1; Figure 1f). All accessions contained six saturated FA (undecylic, palmitic, stearic, arachidic, behenic, and lignoceric acids) and three unsaturated FA (oleic, linoleic and linolenic acids). The dominating acids were palmitic (the percentage in seeds and pods was 21.0% and 29.0%, respectively), linoleic (27.0% in seeds and pods), and linolenic (23.0% in pods) (Figure 4a), which is in line with the data published by other authors [6,38].

The ratio between unsaturated and saturated FA was 2.2 in seeds and 1.4 in pods, i.e., the fatty acid content in seeds was more balanced. When plants respond to abiotic stresses, free FA can play the role of signaling molecules or generate other signaling compounds. For example, linolenic acid participates in the synthesis of such plant hormones as jasmonates. In the human organism, linolenic acid, along with other ω-3 FA, generates bioactive oxylipins—eicosanoids; they play a crucial role in inflammatory processes [39,40,41]. Monoacylgrycerols of palmitic, stearic, and linoleic acids were identified in all accessions. Methylated derivative FA were found only in seeds, whereas hydroxylated ones were found in pods.

#### 2.2.5. Phytosterols

The following phytosterols were identified in both seeds and pods: campesterol, stigmasterol, and β-sitosterol. Isofucosterol and cholesterol were also present in seeds. The total content of sterols was 584 in seeds, and 2972 ppm in pods (Table 1; Figure 1g). The percentage of β-sitosterol in pods was 56; in seeds stigmasterol reached 40% (Figure 4b). Earlier, phytosterols were regarded as compounds reducing the level of cholesterol in blood. More recent studies, however, have shown that the spectrum of their biological activity is much wider, and phytosterol-rich diets contribute to the prevention of cardiovascular and some oncological diseases, liver and intestine disorders, and produce a positive effect on the functions of the immune system [42,43].

#### 2.2.6. Organic Acids

**Figure 5:**
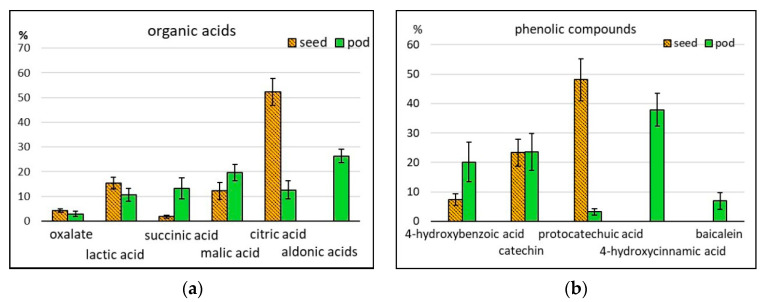
Relative content of some (**a**) organic acids and (**b**) phenolic compounds (% of the total amount of compounds in the given group).

Of all 24 organic acids and their derivatives identified by us, only 12 were present in seeds. Most of them participate in metabolic energy processes (lactic, pyruvic, citric, succinic, fumaric, and malic acids) or in the synthesis of lipids, nucleotides, and phenolic compounds (glyceric, nicotinic, and free phenolic acids). Pods were also found to contain 3-hydroxypropionic, tartaric, 2-ketoglutaric, threonic, erythronic, ribonic, gluconic and saccharic acids, and erythrono-1,4-lactone. The content of acids in seeds and pods was 808 and 2474 ppm, respectively (Table 1; Figure 1h). In pods, 26.5% was the share of aldonic acids, not identified in seeds. Significant differences between seeds and pods were observed in the content of lactic acid (15.3% and 10.6%, respectively), succinic acid (1.8% and 13.2%), and malic acid (12.2% and 19.7%). The greatest difference was found in the content of citric acid: its percentage in seeds was 52.3%, and in pods 12.5% (Figure 5a). One of the main functions of organic acids in a human organism is normalizing the work of the digestive system by regulating the balance between acids and bases, improving digestion processes, producing a beneficial effect on the microbiota in the large intestine, etc.

#### 2.2.7. Phenolic Compounds

Phenolic compounds (PC) are one of the main groups of secondary metabolites in plants. They protect the plant from biotic and abiotic stresses, serve as intermediates in biosynthesis processes, function as structural elements in cell walls, etc. For humans, PC are interesting as bioactive compounds possessing antioxidant, anticancer, and other useful properties [44,45]. The basic PC groups, found in asparagus bean, are phenolic acids and flavonoids. In this research, free phenolic acids were identified (salicylic, protocatechuic, 4-hydroxybenzoic, and 4-hydroxycinnamic) as well as organic acids associated with their metabolism (benzoic and nicotinic). Among flavonoids, flavan-3-ols were present, such as catechin, epicatechin and gallocatechnin, the flavone baicalein, and, among simple phenolics, pyrogallol. The total PC content in seeds was 63, and in pods 263 ppm (Table 1; Figure 1i). Protocatechuic acid dominated in seeds (48.1% of the total PC amount) (Figure 5b), which was confirmed by other authors [4]. In pods, the percentage of 4-hydroxycinnamic acid was 37.9%, but in seeds this compound was absent. The content of catechins in seeds (23.3) and pods (23.5%) was the same. Baicalein was found only in pods (Figure 5b). As far as a broad genetic diversity of PC in cowpea seeds is concerned, it should be taken into consideration that—depending on the employed research methods—the data on the total content of PC, their separate fractions, and their activities vary a lot [46]. A correlation has been observed between the total content of PC in extracts and their antioxidant activity [47,48]. Extracts from whole seeds demonstrated higher antioxidant and anticancer activity than extracts from dehulled seeds. It is associated with the presence of flavonoids, accumulating mostly in seed coat [45,49]. Anticancer activity in asparagus bean may be expressed as the protection of DNA from injuries and suppression of cancer cell proliferation [4,50]. It was noticed in research studies, when the processes undergoing in the intestines were simulated, that different PC fractions, including catechins, were exposed to various degrees of degradation and absorption, but still retained their bioactivity [51,52]. The content and composition of PC in cowpeas as well as their activity change when seeds are soaked, germinated or undergo culinary processing [35,36,50]. The issue of PC activity in a human organism has been actively discussed. PC can function directly or activate enzymes with antimutagenic or anticancer properties [45,52]. Some flavonoids can act as prooxidants, and thus, possibly, participate in the coordination of cell functions. Prooxidant or antioxidant properties of a certain flavonoid depend on its chemical structure and, even more, on its concentration [53]. Some of them, such as catechins, for example, possess the so-called vitamin P activity, affecting the capillary permeability [44]. Catechins are included in the composition of polyphenols, condensed tannins, or proanthocyanidins (PA); they can interact with the human organism as either useful compounds or antinutrients. Their antinutritional properties in many respects depend on the PA molecular weight and structure. Unlike other leguminous plants, asparagus bean contains PA with a high percentage of glycosylated monomers and dimers: the share of compounds with a polymerization degree over 10 is only 13.5% of the total PA. The specific features of PA in asparagus bean could probably smooth their antinutritional effect [52,54].

#### 2.2.8. Saponins

Aglycones of saponins (triterpene glycosides) were identified in asparagus bean seeds and pods (Table 1; Figure 1j). Pods contained aglycones of pentacyclic glycosides–α- and β-amyrins (60 and 294 ppm, respectively); seeds contained cycloartenol, an aglycone of tetracyclic glycosides (110 ppm). On the one hand, saponins are regarded as antinutrients, since they are able to affect the absorption of nutrients in the intestines. On the other, these compounds are considered useful as preventives against cardiovascular and oncological diseases, as they serve as stimulants of the immunity system [55].

#### 2.2.9. Other Compounds

Nucleosides were identified in all accessions. Adenosine was found in both seeds (27) and pods (127); guanosine only in pods (79 ppm) (Tables S1 and S2). Of the compounds participating in different metabolic pathways, urea was identified: its content in seeds was 289 ppm, in pods 446 ppm. A considerable amount of phosphoric acid was also detected in both seeds and pods: 2407 ppm and 1667 ppm, respectively (Table 1). There is nothing surprising in it, because inorganic phosphate plays a crucial role in the energy metabolism and biosynthesis in plants [56].

### 2.3. Results of the Factor Analysis

Factor analysis (principal factors analysis-PFA) was applied to find regularities in the variability and structure of relationships among biochemical characters in asparagus bean seeds and pods.

**Figure 6:**
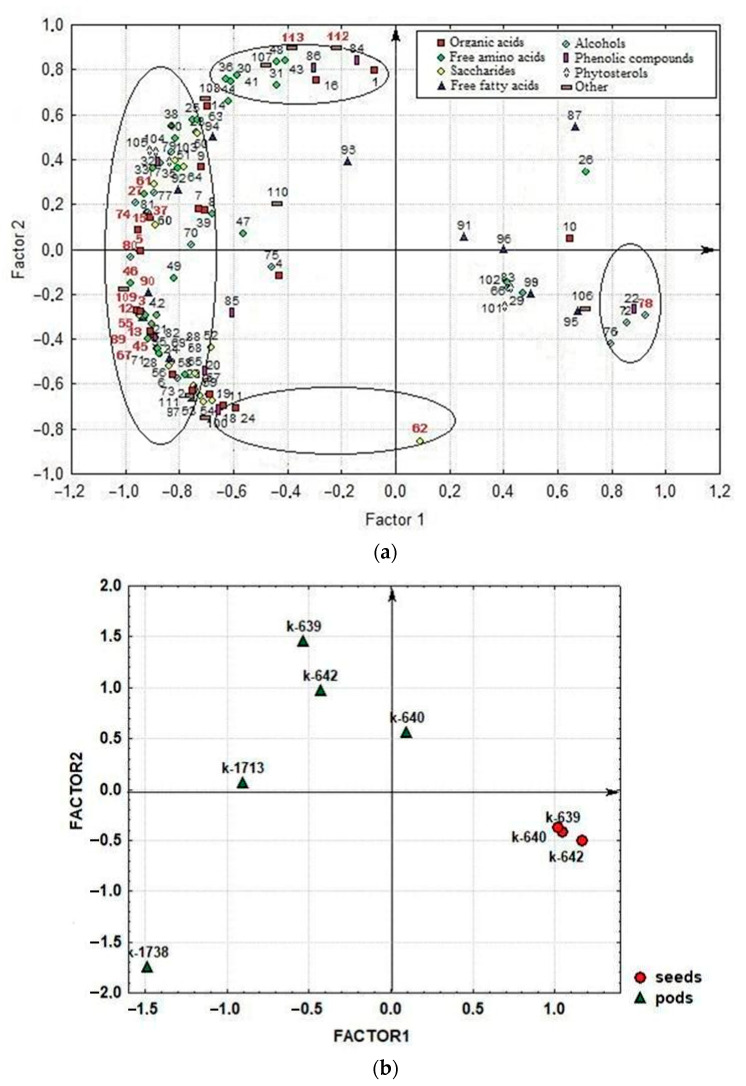
Distribution of the studied compounds and accessions of asparagus bean in the F1–F2 system: (a) factor loading for 113 characters (compounds); (b) factor scores for asparagus bean accessions. Circles indicated correlated groups in negative or positive factor loading. The numbers of the leading traits are marked in red. 1-Oxalate; 2-Lactic acid; 3-3-Hydroxypropionic acid; 4-Pyruvic acid; 5-Glyceric acid; 6-Succinic acid; 7-Malic acid; 8-Fumaric acid; 9-Tartaric acid; 10-Citric acid; 11-2-Ketoglutaric acid; 12-Threonic acid; 13-Erythronic acid; 14-Ribonic acid; 15-Gluconic acid; 16-Saccharic acid; 17-Nicotinic acid; 18-Benzoic acid; 19-Azelaic acid; 20-Salicylic acid; 21-4-Hydroxybenzoic acid; 22-Protocatechuic acid; 23-4-Hydroxycinnamic acid; 24-Erythrono-1,4-lactone; 25-Methyl-phosphate; 26-Phosphoric acid; 27-Valine; 28-Leucine; 29-Isoleucine; 30-Threonine; 31-Methionine; 32-Phenylalanine; 33-Tryptophan; 34-Arginine; 35-α-Alanine; 36-Glycine; 37-Proline; 38-Serine; 39-Tyrosine; 40-Aspartic acid; 41-Asparagine; 42-Glutamic acid; 43-Glutamine; 44-Ornithine; 45-β-Alanine; 46-GABA; 47-Hydroxyproline; 48-Oxoproline; 49-Norleucine; 50-Pipecolic acid; 51-Ornithine lactam; 52-Glycerol-3-phosphate; 53-Ribose; 54-Fructose; 55-Altrose; 56-Sorbose; 57-Galactose; 58-Mannose; 59-Glucose; 60-Rhamnose; 61-Sucrose; 62-Raffinose; 63-Methylglucoside; 64-Glucosamine; 65-Galactose MeOX; 66-Xylitol; 67-Glycerol; 68-Arabinitol; 69-Erythritol; 70-Mannitol; 71-Dulcitol; 72-Sorbitol; 73-Myo-inositol; 74-Ononitol; 75-Galactinol; 76-Methyl-inositol; 77-Desoxyglucitol; 78-Myo-inositol-2-phosphate; 79-Ethanolamine; 80-Phytol; 81-Phytosphingosine; 82-4-Hydroxybenzoic acid; 83-Pyrogallol; 84-Catechin + epi-Catechin; 85-Gallocatechin; 86-Baicalein; 87-Undecylic acid; 88-Palmitic acid; 89-Stearic acid; 90-Arachidic acid; 91-Behenic acid; 92-Lignoceric acid; 93-Oleic acid; 94-Linoleic acid; 95-Linolenic acid; 96-Hydroxybehenic acid; 97-Hydroxylignoceric acid; 98-Linolenic acid-methyl-ester; 99-Lignoceric asid-methyl-ester; 100-Acylglycerols; 101-Cholesterol; 102-Isofucosterol; 103-Campesterol; 104-Stigmasterol; 105-β-Sitosterol; 106-Cycloartenol; 107-α-Amyrin; 108-β-Amyrin; 109-Adenosine; 110-Guanosine; 111-Neophytadiene; 112-Urea; 113-Protein.

The first factor, F_1_ (54.6% of the total variance), encompassed a majority of organic acids, free amino acids, sugars, alcohols, phytosterols, phenolics, and saponins (Figure 6a). Most compounds were interrelated with positive correlations. Some of the compounds (citric, undecylic, protocatechuic and phosphoric acids, sorbitol, methyl-inositol, α-linolenic acid methyl ester, myo-inositol-2-phosphate, and cycloartenol) were negatively correlated with them. The highest factor loading (FL) (> 0.90) was observed for the following biochemical characters: phytol; γ-aminobutyric acid; proline; valine; altrose; glyceric, threonic, gluconic, 3-hydroxypropionic, erythronic, stearic and arachidic acids; sucrose; glycerol; ononitol; phytosphingosine; β-alanine; adenosine; and myo-inositol-2-phosphate. This factor may be interpreted as the factor of general metabolism, as it unites compounds that characterize major metabolic processes in plants. With this, the F_1_ factor disclosed the differences between seeds and pods in the qualitative and quantitative compositions of the identified compounds.

In the second factor, F_2_ (23.3% of the total variance), joint variations were observed in ornithine, methionine, threonine, glycine, asparagine, glutamine, oxoproline, urea, protein, oxalate, saccharic acid, baicalein, catechins, α-amyrin (first group), and raffinose, acylglycerol, 2-ketoglutaric acid, benzoic acid, azelaic acid, and 1,4-lactone (second group) (Figure 6a). The compounds in the first and second groups varied in opposite directions, i.e., the more compounds from the first group were in pods and seeds, the fewer were from the second group. The leading characters within this factor (traits of indicators that have the highest factor loadings, i.e., traits that are most strongly correlated with the factor) (FL > 0.85) were raffinose, urea, and protein. It should be mentioned that high protein content correlated with high contents of compounds from the first group. An interesting dependence was observed: high-protein pods and seeds had a low raffinose content, and vice versa. This factor shows the specific features of metabolism in seeds and pods, associated with different stages of plant ontogenesis. Pods accumulate compounds associated with active substance synthesis: proteins (including enzymes), urea (an intermetabolite in amino acid metabolism), asparagine and glutamine (a nitrogen transport from for protein synthesis), etc. Seeds contain more raffinose and starch—carbohydrates serving as the sources of energy required for seed germination.

The scatterplot of accessions scores within the space of two first factors shows a clear division between seeds and pods in F_1_ (Figure 6b).

Seeds concentrated on the scatterplot to the right, in the area where most of the studied compounds had a low content versus high contents of citric, undecylic, protocatechuic and phosphoric acids, myo-inositol-2-phosphate, sorbitol, methyl-inositol, α-linolenic acid methyl ester, and cycloartenol. Pods settled in the opposite part of the scatterplot; they had higher contents of all compounds, except those having high levels in seeds. In F_2_, seeds grouped in the area with medium contents of compounds correlated within this factor. Compared with seeds, a majority of pods showed higher contents of protein, urea, ornithine, methionine, threonine, glycine, asparagine, glutamine, oxoproline, oxalate, saccharic acid, baicalein, α-amyrin, and catechins versus lower contents of raffinose, acylglycerol, 2-ketoglutaric, benzoic, azelaic acids, and erythrono-1,4-lactone. An exception was the modern cultivar ‘Sibirskiy razmer’: its pods contained more raffinose and less protein than those of other accessions. It should be mentioned that pods of modern cultivars had a lower protein content than those of landraces (k-639, k-640, and k-642), while the content of organic acids, alcohols, monosaccharides, and free fatty acids was higher (Tables S2 and S3). These compounds have a strong effect on the taste and nutritional value of asparagus bean products. It seems that breeding efforts to develop modern cultivars were more concentrated on the improvement of pod taste (organoleptic properties).

## 3. Materials and Methods

### 3.1. Materials

Seeds and pods of asparagus bean accessions from the VIR collection were studied (Table 2). Landraces were collected by N.I. Vavilov during his expedition to China in 1929, while modern cultivars were developed in Russia using selected accessions of Chinese origin in the breeding process. Plant reproductions grown in 2017 at Astrakhan Experiment Stations of VIR were studied (46°070′N, 41°010′E). The climate of the Astrakhan region is dry and sharply continental. The soils in the experimental field were alluvial-meadow, heavy loam, and slightly saline (chloride-sulfate type of salinity). The sum of air temperatures above 10 °C during the vegetation period reached a cumulative 3491.3 growing degree days (°C). The annual amount of precipitation ranged 111.4 mm (http://aisori.meteo.ru/ClimateR; http://meteo.ru/it/178-aisori). The sowing was carried out in a moist, warmed-up soil layer, when the average daily air temperature reached 14–16 °C. Method of sowing is wide-row. Seeds were sown manually, the width between rows was 140 cm, and the distance between seeds in the row was 10 cm. The depth of seeding was 3–5 cm. Accessions were cultivated under irrigated conditions: during the growing season, six irrigations were provided by sprinklers with an average of 250–300 m^3^/ha.

Seeds were harvested for testing in the full ripeness phase. Pods were fixed in the phase of technical ripeness (immature pods), when a pod reaches its maximum size, but remains green, and its seeds just start to ripen. The harvested greed pods were dried first in a thermostat at 24 °C, then in open air under a tent. An average seed sample weighed no less than 50 g was ground in Lab.mill-1 QC-114. Pods were collected from 10 plants, and the dried material was ground in a coffee grinder.

### 3.2. Chemicals and Reagents

Pyridine, tricosane, *N*,*O*-bis(trimethylsilyl)trifluoroacetamide were obtained from Sigma-Aldrich (St. Louis, MO, USA), catalyst Kjeltabs was obtained from Foss (Höganäs, Sweden), and all other chemicals were of analytical grade.

### 3.3. Protein Determination

The biochemical analysis was performed according to VIR’s guidelines [57].The protein content was measured using the Kjeldahl method. Three hundred mg of an accession (3–4 samples) was mineralized with 5 mL of concentrated sulfuric acid and 1.1 g catalyst Kjeltabs at 420 °C for 90 min; nitrogen was distilled on a Foss Kjeltec 2200 Auto Distillation Unit (Höganäs, Sweden), with subsequent titration with 0.1 N solution of sulfuric acid; total proteins were calculated from the nitrogen content using factor 6.25. The values were expressed as ‘%/dry matter’. The predetermined content of dry matter in seeds and pods was 92.3% and 92.4%, respectively.

### 3.4. GC-MS Analysis

Primary and secondary metabolites were analyzed using gas chromatography with mass spectrometry according to a published protocol [58]. The ground sample (40–50 mg) was mixed with methanol at a ratio of 1:10 (*w*:*v*); the sample was infused for no less than 30 days at 5–6 °C; the resulting extract was centrifuged at 14,000 rpm for 10 min;100 μL of the extract was dried on a CentriVap Concentrator (Labconco, Kansas, MI, USA); then 20 μL of tricosane solution in pyridine (concentration: 1 μg/μL) was added to the sample as an internal standard, and the result was silylated with 50 µL of *N*,*O*-bis(trimethylsilyl)trifluoroacetamide for 40 min at 100 °C. The sample (1.2 μL) was separated using an HP-5MS capillary column (5% phenyl 95% methylpolysiloxane, 30.0 m, 250.00 μm, 0.25 μm; Agilent Technologies, Palo Alto, CA, USA) on an Agilent 6850 gas chromatograph with a quadrupole mass selective detector (Agilent 5975B VL MSD, Agilent Technologies). Conditions of the analysis: inert gas flow in the column 1.5 mL/min; temperature program from +70 °C up to +320 °C, with heating rate 4 °C/min; evaporator temperature +300 °C, flow division ratio 1:20. The chromatogram was registered in the full ion flow scan mode at 2.0 scans per second. Ionization by electron impact was performed at 70 eV, with the ion source temperature 230 °C. The recording of a chromatogram started after 4 min required for solvent removal and went on for 62 min. Compounds were identified using AMDIS software (Version 2.69, National Institute of Standards and Technology, USA, http://www.amdis.net). Libraries used in the process of analysis: NIST 2010 (National Institute of Standards and Technology, USA, http://www.nist.gov), and the collections of standard compound mass spectra maintained by St. Petersburg State University and the Komarov Botanical Institute [59,60]. These last two databases were developed as the result of previous standard-based chemical determination performed at St. Petersburg University and the Botanical Institute of the Russian Academy of Sciences. The retention indices (RI) were estimated by the calibration of saturated hydrocarbons with the number of C atoms ranging from 10 to 40. A semi-quantitative assay of the metabolite profiles was performed by calculation of the total ion peak areas with the internal standard method using UniChrom software (UniChrom TM 5.0.19.1134, New Analytic Systems LLC, Belarus, www.unichrom.com). The content of the identified compounds was expressed in ‘ppm (mg/kg)/dry mattert’.

### 3.5. Statistical Analysis

Statistical data processing was based on MS Excel 2007 and Statistica 7.0 software. Interrelations among the identified biochemical characters were analyzed using factor analysis (principal factors analysis-PFA) [61].

## 4. Conclusions

The results presented here demonstrate potential nutritional value of seeds and pods yielded by old Chinese landraces of asparagus bean and its modern cultivars ‘Yunanskaya’ and ‘Sibirskiy razmer’ developed on the basis of genotypes from China. Biochemical compositions of seeds and pods were diverse. According to the data obtained by metabolomic profiling, 120 compounds have been identified in total, part of them being bioactive. Most of such bioactive compounds are the so-called ‘secondary metabolites’, as these substances do not have their own pathways of synthesis and use for their formation basic metabolic pathways of plants. Such division is conditional, since the pathways of primary and secondary metabolisms are often difficult to tell apart. The identified variations in the biochemical compositions of seeds and pods reflect their ‘specialization’, i.e., different stages of plant development and different levels of metabolism corresponding to them. The function of seeds is to preserve such compounds that ensure germination of the future plant. Pods in the phase of ‘milk ripeness’ are characterized by intensive metabolism; therefore, the content of a majority among the identified metabolites in them is significantly higher than in seeds. Provided that these differences are taken into account, it would be easier to include asparagus bean seeds or pods in various diets with maximum efficiency. Some of the compounds present in asparagus bean are numbered among antinutrients; however, certain culinary processing techniques make it possible to minimize their negative effect. Some of the differences observed in the composition of pods collected from Chinese landraces and from modern cultivars suggest the trend taken by the breeding practice in the development of these cultivars. Landraces had a higher content of proteins, while modern cultivars contained more organic acids, polyols, monosaccharides, and free fatty acids. The content of phenolics, phytosterols, and saponins depended on individual features of a variety: accessions with higher contents of these groups of compounds were found both among landraces and commercial cultivars. Further research, encompassing a greater number of accessions, would generate new data on the tendencies uncovered by this study, so that they could be used to develop dietary recommendations.

## Figures and Tables

**Table 1:** Average total content of the main groups of metabolites and its variability in seeds and pods of *Vigna unguiculata*.

Metabolites	Seeds		Pods	
	Mean ± SE	Min–Max	Mean ± SE	Min–Max
Protein*	27.10 ± 0.22	26.75–27.51	29.98 ± 1.10	26.90–33.50
Free amino acids**	433 ± 69	340–567	20,433 ± 2310	14,016–25,727
Total saccharides**	15,596 ± 1574	12,720–18,142	37,072 ± 4032	27,331–50,046
Oligosaccharides**	15,522 ± 1568	12,669–18,075	32,602 ± 2066	26,500–38,462
Monosaccharides**	74 ± 16	51–105	4409 ± 2625	807–14,764
Polyols**	1127 ± 125	897–1327	5678 ± 1557	2822–11,110
Free fatty acids**	1827 ± 213	1472–2209	3588 ± 897	1992–6807
Phytosterols**	584 ± 113	400–790	2972 ± 307	1954–3785
Organic acids**	808 ± 143	634–1093	2474 ± 447	1495–3867
Phenolic compounds**	63 ± 9	48–79	263 ± 30	193–364
Saponins**	110 ± 48	43–204	354 ± 81	212–643
Phosphoric acids**	2407 ± 240	2127–2885	1667 ± 236	1037–2274
Urea**	289 ± 15	271–319	446 ± 102	181–780

*—content in %/dry matter, **—content in ppm (mg/kg)/dry matter.

**Table 2:** Material for the research.

No.	VIR Catalogue Number	Accession Name	Origin	Year of Acquisition	Comments
**1**	k-639	Landrace	China	1929	N.I. Vavilov’s expedition to China
**2**	k-640	Landrace	China	1929	N.I. Vavilov’s expedition to China
**3**	k-642	Landrace	China	1929	N.I. Vavilov’s expedition to China
**4**	k-1713	Cv. ‘Yunanskaya’	Novosibirsk Province, Russia	2006	
**5**	k-1738	Cv. ‘Sibirskiy razmer’	Novosibirsk Province, Russia	2006	

## Supplementary Materials

The following are available online. Table S1: The content of primary and secondary metabolite compounds in *Vigna unguiculata* (L.) Walp. seeds; Table S2: The content of primary and secondary metabolite compounds in *Vigna unguiculata* (L.) Walp. pods of old Chinese landraces and the modern cultivars ‘Yunanskaya’ and ‘Sibirskiy razmer’; Table S3: The content of primary and secondary metabolite compounds in *Vigna unguiculata* (L.) Walp. pods of old Chinese landraces.
