# Supplementary material for: Composition of Primary and Secondary Metabolite Compounds in Seeds and Pods of Asparagus Bean (Vigna unguiculata (L.) Walp.) from China
Source: Molecules. 2020 Aug 19;25(17):3778. doi: 10.3390/molecules25173778 (PMC7503259; doi:10.3390/molecules25173778)
Supplement: Supplementary file 1 [file molecules-25-03778-s001.pdf]

molecules

MDPI

Supplementary Materials for

Composition of Primary and Secondary Metabolite Compounds in Seeds and Pods of Asparagus Bean (*Vigna unguiculata* (L.) Walp.) from China

Table S1. The content of primary and secondary metabolite compounds in *Vigna unguiculata* (L.) Walp. seeds (ppm (mg/kg)/ dry matter).

Metabolite compound	Mean	Minimum	Maximum	Standard Error
Oxalate	35	22	57	11
Lactic acid	120	85	150	19
Pyruvic acid	22	10	30	6
Glyceric acid	18	18	18	0
Succinic acid	13	6	20	4
Malic acid	95	56	133	22
Citric acid	436	268	665	119
Nicotinic acid	10	6	16	3
Azelaic acid	34	17	45	9
4-Hydroxybenzoic acid	4	3	6	2
Protocatechuic acid	31	21	46	8
Fumaric acid	6			
Valine	9	5	15	3
Leucine	6	4	7	1
Isoleucine	2			
Threonine	18	5	43	12
Phenylalanine	79	30	111	25
Tryptophan	21	4	37	9
α-Alanine	16	10	23	4
Glycine	62	21	98	22
Proline	10	5	15	5
Serine	16			
Tyrosine	9	5	11	2
Aspartic acid	57	29	84	28
Asparagine	42	11	67	17
Glutamic acid	50	39	57	5
Oxoproline	62	30	95	19
Ornithine lactam	12	6	15	3
Glycerol-3-phosphate	12	11	13	1
Fructose	15	9	24	5
Sorbose	9	6	13	2
Mannose	19	10	27	5
Glucose	20	15	29	4
Sucrose	13509	11807	15281	1003
Raffinose	2013	862	2794	588
Xylitol	6			
Glycerol	112	80	145	19
Arabinitol	19	6	39	10
Sorbitol	55	39	67	8
Myo-inositol	216	73	448	117
Galactinol	452	373	599	74
Methyl-inositol	205	164	242	23

**Myo-inositol-2-phosphate**	65	58	77	6
**4-Hydroxybenzoic acid**	4	3	6	2
**Protocatechuic acid**	31	21	46	8
**Nicotinic acid**	10	6	16	3
**Pyrogallol**	15			
**Catechin+epi-Catechin**	14	11	19	3
**Undecylic acid**	26	21	32	3
**Palmitic acid**	359	261	471	61
**Stearic acid**	94	91	97	2
**Arachidic acid**	17	16	18	1
**Behenic acid**	45	26	75	15
**Lignoceric acid**	20	15	27	4
**Lignoceric asid-methyl-ester**	20	19	21	1
**Oleic acid**	245	65	502	132
**Linoleic acid**	469	322	701	117
**Linolenic acid**	162	95	228	66
**Linolenic acid-methyl-ester**	431	48	1117	344
**Acylglycerols**	129	111	153	12
**Cholesterol**	23			
**Isofucosterol**	162			
**Campesterol**	107	31	182	44
**Stigmasterol**	217	195	232	12
**β-Sitosterol**	198	151	223	23
**Cycloartenol**	110	43	204	48
**Adenosine**	27	6	50	13
**Urea**	289	271	319	15
**Methyl-phosphate**	8	4	11	2
**Phosphoric acid**	2407	2127	2885	240
**Protein***	27,10	26,75	27,51	0,22

* - content in %/ dry matter

Table S2. The content of primary and secondary metabolite compounds in *Vigna unguiculata* (L.) Walp. pods of old Chinese landraces and the modern cultivars 'Yunanskaya' and 'Sibirskiy razmer' (ppm (mg/kg)/ dry matter).

Organic compound	Mean	Minimum	Maximum	Standard Error
**Oxalate**	57	10	85	14
**Lactic acid**	294	134	808	130
**3-Hydroxypropionic acid**	21	10	39	5
**Pyruvic acid**	26	22	30	1
**Glyceric acid**	53	20	75	10
**Succinic acid**	397	78	1055	183
**Malic acid**	472	272	9209	117
**Fumaric acid**	7	4	10	1
**Tartaric acid**	14	6	26	4
**Citric acid**	257	101	367	52
**2-Ketoglutaric acid**	23			
**Threonic acid**	171	34	338	50
**Erythronic acid**	55	15	110	17
**Ribonic acid**	40	2,18	6,51	0,79
**Gluconic acid**	350	206	501	55
**Saccharic acid**	22	15	28	6
**Nicotinic acid**	24	17	30	2
**Benzoic acid**	13			
**Azelaic acid**	50	23	112	17

**4-Hydroxybenzoic acid**	51	27	115	16
**Protocatechuic acid**	7	4	9	3
**4-Hydroxycinnamic acid**	104	59	162	24
**Erythrono-1,4-lactone**	28			
**Valine**	720	504	912	79
**Leucine**	112	27	301	49
**Threonine**	313	150	545	64
**Methionine**	58	9	96	14
**Phenylalanine**	616	401	873	79
**Tryptophan**	420	351	538	31
**Arginine**	63	17	153	24
**α-Alanine**	951	783	1147	68
**Glycine**	3705	1333	5684	841
**Proline**	4889	3665	7372	672
**Serine**	1069	801	1349	97
**Tyrosine**	262	142	560	76
**Aspartic acid**	212	152	308	26
**Asparagine**	2598	817	4892	712
**Glutamic acid**	239	130	427	55
**Glutamine**	1002	147	1955	367
**Ornithine**	140	32	218	30
**β-Alanine**	66	25	131	21
**GABA**	1493	828	2481	310
**Hydroxyproline**	234	197	272	37
**Oxoproline**	733	137	1584	236
**Norleucine**	268	238	290	16
**Pipecolic acid**	19	14	26	2
**Ornithine lactam**	500	233	786	109
**Glycerol-3-phosphate**	249	51	404	104
**Ribose**	52	3	174	32
**Fructose**	2090	280	7451	1360
**Altrose**	23	11	47	6
**Sorbose**	490	128	1325	213
**Galactose**	53	10	199	37
**Mannose**	728	162	2207	374
**Glucose**	813	155	3051	561
**Rhamnose**	10	6	16	2
**Sucrose**	31519	25953	37615	1907
**Raffinose**	1083	320	3249	548
**Methylglucoside**	24	17	37	4
**Glucosamine**	17	6	29	4
**Galactose MeOX**	49	47	51	2
**Glycerol**	9848	499	1973	279
**Arabinitol**	218	113	490	71
**Erythritol**	100	38	238	36
**Mannitol**	753	341	1761	264
**Dulcitol**	1330	549	3096	458
**Myo-inositol**	1338	358	3261	569
**Ononitol**	29	16	35	3
**Galactinol**	624	240	906	109
**Methyl-inositol**	37	13	57	7
**Desoxyglucitol**	55	39	83	8

**Myo-inositol-2-phosphate**	11	8	15	3
**Ethanolamine**	65	44	101	10
**Phytol**	104	80	157	14
**Phytosphingosine**	37	10	51	8
**Salicylic acid**	16	14	18	2
**4-Hydroxybenzoic acid**	51	27	115	16
**Protocatechuic acid**	7	4	9	3
**4-Hydroxycinnamic acid**	104	59	162	24
**Catechin+epi-Catechin**	67	17	108	21
**Gallocatechin**	21	13	29	8
**Baicalein**	17	5	24	6
**Undecylic acid**	30	24	35	3
**Palmitic acid**	1009	609	1787	234
**Stearic acid**	308	209	528	58
**Arachidic acid**	49	15	72	10
**Behenic acid**	36	25	48	4
**Lignoceric acid**	66	32	98	13
**Hydroxybehenic acid**	8			
**Hydroxylignoceric acid**	33	17	67	9
**Oleic acid**	147	85	226	25
**Linoleic acid**	1016	412	2194	319
**Linolenic acid**	906	336	1987	305
**Acylglycerols**	196	55	439	69
**Campesterol**	336	219	444	37
**Stigmasterol**	956	743	1265	98
**β-Sitosterol**	1681	980	2076	195
**α-Amyrin**	60	16	121	20
**β-Amyrin**	294	196	523	62
**Adenosine**	127	46	250	34
**Guanosine**	79	54	104	25
**Neophytadiene**	94	73	115	21
**Urea**	446	181	780	102
**Methyl-phosphate**	20	16	27	2
**Phosphoric acid**	1667	1037	2274	236
**Protein***	29,98	26,90	33,50	1,10

* - content in %/ dry matter

Table S3. The content of primary and secondary metabolite compounds in *Vigna unguiculata* (L.) Walp. pods of old Chinese landraces (ppm (mg/kg)/ dry matter).

Organic compound	Mean	Minimum	Maximum	Standard Error
**Oxalate**	71	47	85	12
**Lactic acid**	145	134	152	6
**3-Hydroxypropionic acid**	14	10	20	3
**Pyruvic acid**	24	22	25	1
**Glyceric acid**	39	20	51	10
**Succinic acid**	135	78	245	55
**Malic acid**	359	272	480	63
**Fumaric acid**	6	4	10	2
**Tartaric acid**	16	7	26	5
**Citric acid**	284	168	367	60
**Threonic acid**	102	34	137	34

**Erythronic acid**	33	15	57	12
**Ribonic acid**	46	25	65	12
**Gluconic acid**	302	206	441	71
**Saccharic acid**	22	15	28	6
**Nicotinic acid**	23	17	30	4
**Azelaic acid**	28	23	36	4
**4-Hydroxybenzoic acid**	32	27	39	4
**Protocatechuic acid**	7	4	9	3
**4-Hydroxycinnamic acid**	99	67	162	32
**Leucine**	49	27	61	11
**Threonine**	383	301	545	81
**Methionine**	74	62	96	111
**Phenylalanine**	564	401	668	83
**Tryptophan**	429	351	538	56
**Arginine**	33	17	48	9
**α-Alanine**	912	783	1076	86
**Glycine**	4471	2354	5684	1062
**Proline**	3977	3665	4426	230
**Serine**	1130	906	1349	128
**Tyrosine**	189	142	227	25
**Aspartic acid**	225	152	308	45
**Asparagine**	3182	1444	4892	996
**Glutamic acid**	183	130	284	50
**Glutamine**	1498	766	1955	370
**Ornithine**	149	137	161	7
**β-Alanine**	32	25	44	6
**GABA**	1025	828	1299	141
**Hydroxyproline**	272			
**Oxoproline**	986	636	1584	300
**Norleucine**	238			
**Pipecolic acid**	21	14	26	4
**Ornithine-lactam**	452	233	728	146
**Glycerol-3-phosphate**	51			
**Ribose**	8	3	13	3
**Fructose**	464	280	637	103
**Altrose**	15	10	18	2
**Sorbose**	243	128	311	58
**Galactose**	13	10	18	2
**Mannose**	309	162	402	74
**Glucose**	205	155	278	37
**Rhamnose**	10	7	14	2
**Sucrose**	29344	25953	32439	1878
**Raffinose**	439	320	547	66
**α-Methylglucoside**	28	19	37	5
**Glucosamine**	15	6	26	6
**Glycerol**	575	499	682	55
**Arabinitol**	124	113	146	11
**Erythritol**	54	38	70	9
**Mannitol**	416	341	565	75
**Dulcitol**	781	549	1085	159
**Myo-inositol**	467	358	618	78
**Ononitol**	26	16	32	5

**Galactinol**	658	614	736	39
**Methyl-inositol**	26	13	35	7
**Desoxyglucitol**	47	39	58	6
**Myo-inositol-2-phosphate**	11	8	15	3
**Ethanolamine**	59	44	70	8
**Phytol**	85	80	92	4
**Phytosphingosine**	31	10	51	12
**4-Hydroxybenzoic acid**	32	27	39	4
**Protocatechuic acid**	7	4	9	3
**4-Hydroxycinnamic acid**	99	67	162	32
**Catechin+epi-Catechin**	83	49	109	18
**Baicalein**	17	5	24	6
**Undecylic acid**	30	24	35	3
**Palmitic acid**	647	609	682	21
**Stearic acid**	243	209	297	27
**Arachidic acid**	34	15	47	10
**Behenic acid**	39	31	48	5
**Lignoceric acid**	68	32	98	19
**Hydroxybehenic acid**	8			
**Hydroxylignoceric acid**	38	21	67	15
**Oleic acid**	157	116	226	35
**Linoleic acid**	594	412	848	131
**Linolenic acid**	478	336	736	129
**Acylglycerols**	96	55	139	24
**Campesterol**	333	219	444	65
**Stigmasterol**	1026	755	1265	148
**β-Sitosterol**	1683	980	2076	352
**α-Amyrin**	85	43	121	23
**β-Amyrin**	352	203	523	93
**Adenosine**	87	46	115	21
**Guanosine**	54			
**Urea**	563	354	780	123
**Methyl-phosphate**	21	16	27	3
**Phosphoric acid**	1959	1448	2274	258
**Protein***	31,33	29,50	33,50	1,17

* - content in %/ dry matter
